# Microfluidics Approach to the Mechanical Properties of Red Blood Cell Membrane and Their Effect on Blood Rheology

**DOI:** 10.3390/membranes12020217

**Published:** 2022-02-13

**Authors:** Claudia Trejo-Soto, Guillermo R. Lázaro, Ignacio Pagonabarraga, Aurora Hernández-Machado

**Affiliations:** 1 Instituto de Física, Pontificia Universidad Católica de Valparaiso, Casilla 4059, Chile; 2 Departament de Física de la Materia Condensada, Universitat de Barcelona, Av. Diagonal 645, 08028 Barcelona, Spain; grolazaro@gmail.com (G.R.L.); ipagonabarraga@ub.edu (I.P.); a.hernandezmachado@gmail.com (A.H.-M.); 3 CECAM, Centre Europeén de Calcul Atomique et Moleéculaire, École Polytechnique Feédeérale de Lausanne (EPFL), Batochime—Avenue Forel 2, 1015 Lausanne, Switzerland; 4 Universitat de Barcelona Institute of Complex Systems (UBICS), Universitat de Barcelona, 08028 Barcelona, Spain; 5 Centre de Recerca Matemàtica, Edifici C, Campus de Bellaterra, 08193 Barcelona, Spain; 6 Institute of Nanoscience and Nanotechnology (IN2UB), University of Barcelona, 08028 Barcelona, Spain

**Keywords:** membrane elasticity, red blood cells, hemodynamics, hemorheology, microfluidics

## Abstract

In this article, we describe the general features of red blood cell membranes and their effect on blood flow and blood rheology. We first present a basic description of membranes and move forward to red blood cell membranes’ characteristics and modeling. We later review the specific properties of red blood cells, presenting recent numerical and experimental microfluidics studies that elucidate the effect of the elastic properties of the red blood cell membrane on blood flow and hemorheology. Finally, we describe specific hemorheological pathologies directly related to the mechanical properties of red blood cells and their effect on microcirculation, reviewing microfluidic applications for the diagnosis and treatment of these diseases.

## 1. Introduction

The membrane is a fundamental structure in all living organisms, as it defines the cell as an entity. It separates the external environment from the cell’s inner region, which contains all the organelles and molecular machinery. The elastic behavior of more complex membranes, such as those present in mammalian cells, is still subject to lively debate in the literature. In this context, most research has focused on the study of human red blood cells as a mechanical model system [1,2], due to its structural simplicity and the lack of a nucleus and any internal structure.

From a theoretical point of view, our knowledge about membranes’ molecular compositions and functioning has continuously increased from the pioneer biological model of the fluid mosaic by Singer and Nicolson [3]. In the last 40 years, membranes have also been studied by physicists, providing a complementary picture about membrane behavior and the properties of vesicles and cells. The subject was first approached by Canham in 1970 [4] and then by Helfrich in 1973 [5,6], and based on their models an outstanding number of membrane phenomena have been understood and explained from a physical perspective. Additionally, in spite of membranes’ intrinsic complexity, physical models have explained a high number of phenomena observed experimentally, inviting an extensive theoretical exploration of biological membranes.

Erythrocytes or red blood cells (RBCs hereafter) present a remarkable capability to deform and pass through very thin capillaries, and in microcirculation they acquire strange shapes, the benefits of which are still unknown; see the article of G. Tomaiuolo 2009 [7]. The dynamics of RBCs in shear flow have been studied and an unsteady tumbling solid-like motion has been observed when cells are suspended in plasma [8]. Additionally, at high shear stress they exhibit a drop-like tank-treading motion characterized by a steady orientation and membrane rotation about the internal fluid [9,10,11]. They also develop a number of different morphologies if their membranes are altered or damaged, as known from a number of anemias, malaria, or during blood storage [12]. The delicate membrane equilibrium at the molecular scale ultimately affects the mechanisms taking place at a much larger scale, such as cell shape and blood properties.

Initially, RBCs were studied from a numerical point of view, considering confined geometries to simulate the circulatory system conditions. In the present decade, the developments in microfluidics technologies have contributed significantly to the experimental study of RBCs’ membrane properties [13] and the rheological properties of blood and its relation to the RBCs [14,15]. The combination of biological studies with microfluidics has been fundamental in the biomedical research of the biomechanical properties of the RBCs in health and disease [16] and the development of new Point of Care Diagnostics (PoCD) techniques using blood [17,18,19].

Considering that the blood is the most important fluid in our body and that blood circulation plays a fundamental role in maintaining an appropriate environment in the body’s tissues, ensuring the optimal functioning of cells, understanding the flow properties of blood is crucial. These properties depend on the composition of blood and the particular properties of its constituents. The blood is known to be a complex mixture of blood plasma and blood cells. It presents a non-Newtonian behavior, even if blood plasma behaves as a Newtonian fluid by itself. The study of blood flow can be approached from two points of view: we can study the fluid dynamics of the blood flow as a continuous fluid with its constitutive equations or we can study the flow properties and rheology of blood and its components’ contributions, from single cells to their collective behavior.

This review is dedicated to describing the general features of RBC membranes and their effect on blood flow, using numerical and experimental studies based on microfluidics technologies. Section 2 is dedicated to describing the composition of the cell membrane in order to understand its constitution. In Section 3, we discuss current numerical modeling techniques of RBC membranes. In Section 4, we review the composition of blood, and the mechanical properties of RBCs. Here, we define how these properties affect the behavior of blood and its consequences. In Section 5, we refer to the past and current studies of the characteristics of RBCs and blood flow at the microscale and their effect on blood rheology from a single cell to the collective behavior. Finally, Section 6 is dedicated to describing our interest in hemorheology, exposing its high importance in the diagnosis of diseases related to blood viscosity and the properties of RBC membranes, focusing on novel microfluidics applications for diagnosis and treatment.

## 2. Cell Membranes

Cell membranes represent an essential element in the development of living organisms. They constitute the cells’ boundaries, separating the interior of the cell from the external environment. Membranes enclose the organelles and components that together form the basic units of life. However, membrane functionality is not limited to its simple structural role, but membranes are also responsible for the interactions of the cell with neighboring cells. These interactions are mediated by a certain type of transmembrane proteins that coordinate the cell signaling, enabling the cell’s response to environmental pressures. Additionally, membranes maintain ion gradients which allow the synthesis of ATP, the basic energetic molecule [20]. The plasma membrane is the most important membrane of the cell, but other types of membranes are present in organelles such as the nucleus, the Golgi apparatus, the endoplasmic reticulum and the mitochondria. All the membranes of the cell constitute around 30% of the total protein activity [21].

All biological membranes share a common structure and composition in spite of being part of different entities, and regardless of their function. Membranes are composed of different lipid molecules that assemble to form bilayers. Lipid bilayers are selectively permeable to the exchange of polar molecules and host a high density of transmembrane proteins, which essentially define the specific membrane functionality. Lipids are bound by relatively weak, non-covalent interactions that allow a rapid lateral interchange of positions, leading to a significant surface diffusion over the membrane plane, $D\approx 10^{-12}$$m^{2}$/s, refs. [12,22]. The lipids practically behave as a fluid in the bilayer plane, a property with important implications for the cell activity. The membrane is connected with the inner cytoskeleton, a three-dimensional mesh formed by actin filaments which provides compactness and structural ordering, and determines the cell shape, which in turn depends on the type of cell and its function. In some cells, an exterior cytoskeleton also exists, and it connects with neighboring cells in order to facilitate a coordinate response of the tissue. The membrane equilibrium is controlled by a number of active processes, including a flip-flop rearrangement of the different lipid species of the bilayer, remodeling of the cytoskeleton, or the balance of lipid densities during vesiculation processes (e.g., during endo- and exocytosis), which are able to occur due to the existence of lipid reservoirs in the interior of the cell.

**Figure 1:**
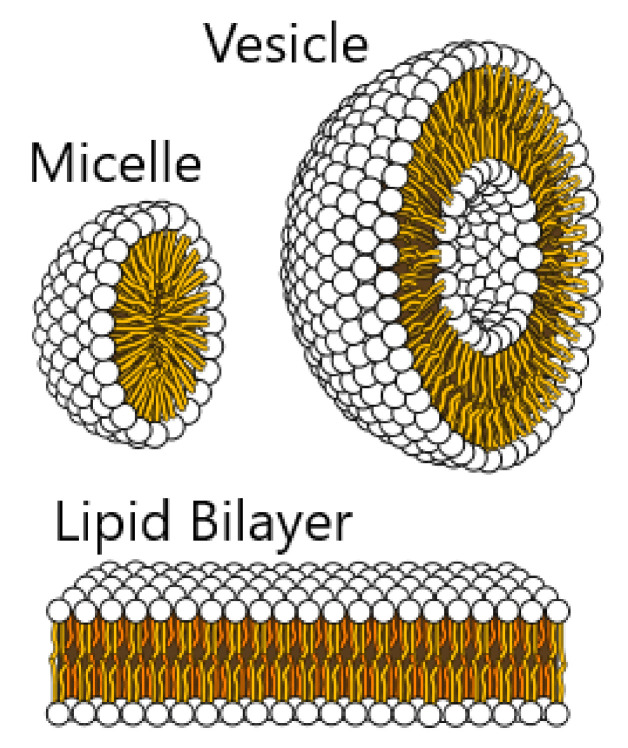
Different aggregates formed by lipids: micelle, bilayer, and closed bilayer, forming a vesicle. The preference of the lipids to aggregate in one structure or another is determined by the shape of the lipid; phospholipids form bilayers. Credits: Mariana Ruiz Villareal available under Public Domain.

Lipids represent up to 50% of the total mass of the membrane in mammalian cells [23,24]. They are amphiphilic molecules with a polar head (which prefers to contact and interact with other polar molecules, such as water) and a tail formed by two hydrocarbon chains which present a strong hydrophobicity, and therefore the tails avoid the interacting with water. If lipids are immersed in water, they tend to self-assemble to avoid the hydrophobic interactions with the surrounding water. Two basic structures can be formed by these aggregates. Sometimes they assemble to form micelles, a closed structure, with all the tails in the inner, free-water region, and the lipid heads oriented to the exterior, in contact with water. Another possibility is the formation of bilayers and vesicles, when two lipid monolayers fold in opposite directions, so that the heads form two parallel sheets whereas the tails are trapped in the intermediate region, without contact with the aqueous environment, see Figure 1. Lipids rearrange to avoid the presence of edges, forming closed surfaces in which the water is at both the inner and outer regions, but there is no direct interaction with the tails. The strong hydrophobicity causes these closed structures to be much energetically favorable, thus ensuring large stability under thermal fluctuations and other mechanical disruptions [23].

A eukaryotic cell is typically composed of 500–1000 different species of lipids; however, the major components reduce to the phospholipids, which are asymmetrically distributed in the bilayers. A discussion on the polymorphism of lipids can found in the work of Cullis (1986) [25]. In addition to the phospholipids, animal cell membranes also contain cholesterol and glycolipids. Cholesterol is a small molecule with a polar hydroxyl group and a short hydrocarbon chain. Cholesterol occupies the space between phospholipid tails in the inner region of the bilayer, with its head oriented close to the phospholipid head. Mammalian cell membranes are rich in cholesterol, which plays an important role in the control of bilayer fluidity, and it also affects the membrane rigidity when present at abnormally high densities [26,27]. Another important constituent of the cell membrane is transmembrane proteins, responsible for the main processes that take place in the membrane, and therefore they define the membrane functionality. Depending on the membrane, proteins represent 25–75% of the total mass of the membrane. Since proteins are much larger than lipids, this concentration corresponds to a protein per ≈50–100 lipids. Transmembrane proteins are also amphiphilic and orient their polar groups to the aqueous environment (cytosol and exterior of the cell), whereas the hydrophobic groups interact with the lipid tails. The bilayers of mammalian cells are complex structures with a bewildering number of proteins working on and through them. They have a typical thickness of 4 nm, while most eukaryotic cells are ≈5 μm–8 μm in length. Thus, the membrane thickness is three orders of magnitude smaller than the overall cell length. Although the bilayer is usually fluid, this property presents a strong dependence on the temperature and lipid composition [28].

Most cells have a complex mesh formed by actin filaments that occupies most of the inner cytosolic volume and connects the different organelles and microstructures of the cell. This structural element provides mechanical strength to the cell and it often participates in determining the cell shape and cell mobility. This structure, known as a cortical cytoskeleton, is connected with the membrane in order to coordinate the response to external perturbations [29]. The cells also contains a much simpler cytoskeletal structure, the so-called membrane cytoskeleton, which lies underneath the lipid bilayer. The membrane cytoskeleton has a structural functionality, providing strength and preventing from certain shape deformations, such as vesiculation or the pinching-off of the bilayer. The membrane cytoskeleton is a two-dimensional spectrin network anchored to the inner (cytosolic) monolayer of the plasma bilayer of certain cells [30], such as human erythrocytes. The presence of ATP is crucial for maintaining the cytoskeleton properties, and when this molecule is depleted, the cell experiences drastic shape changes. Although this phenomenon is not completely understood, the fluid gel hypothesis assumes that the network is subjected to continuous remodeling, which allows the relaxation of cytoskeleton tensions [31]. Hence, when active processes cease, the cytoskeleton loses its fluidic behavior and stiffens.

Eukaryotic cells present an extensive variety of shapes, as an adaptation to their specific function and location within the different tissues. The cortical cytoskeleton and the plasma membrane are the two main elements responsible for the cell shape and mechanical response. Still, the different organelles occupy an important portion of the cell volume, and their presence implies that the cell must accommodate them. Hence, while studying the mechanical properties of the cell, it is difficult to discern between the different effects, obscuring the understanding of the specific properties of the membrane. Taking into account this problem, the RBC represents an interesting case. Mammalian RBCs lack a nucleus and any internal structure, so that their unique components are the plasma membrane with its underlying cytoskeleton [32]. Accordingly, the shape of the RBC can be directly understood as the result of its membrane properties. The RBC is therefore studied as a model system in order to understand plasma membrane properties and, indeed, many of the studies that have elucidated key insights on membrane biology focused on RBCs. Nevertheless, RBCs are interesting not only as a model system but also due to their crucial role in our lives, as they are the main component of blood and the unique carriers of oxygen.

## 3. Cell Membrane Modeling

To develop a physical approach to membrane modeling, the use of mesoscopic theories is beneficial. Considering the membrane as locally homogeneous and introducing a continuum description, each small part of the membrane is characterized by some certain local properties. These properties must be consistent with the local molecular structure of the membrane, so that a connection between the micro and meso scales can be derived. The molecular complexity of biological membranes only affects a few essential aspects of relevance in a physical description of membranes: length scale separation, fluidity, hydrophobicity of the lipid tails, bilayer architecture, membrane cytoskeleton and active processes [33].

In this context, the Helfrich bending energy represents the fundamental theory of membrane elasticity. Helfrich adapted the general theory of elasticity to the particular characteristics of membranes, accounting for the structural membrane properties [5]. The main assumption of this approach is that the cell membrane can be described as a two-dimensional sheet, based on its small thickness compared to the cell length. Helfrich proposed that, from the main types of deformations that a layer can undergo—shear, tilt, stretch and bending—only the latter plays a relevant role in the membrane elasticity to characterize the shape of the RBC. He generalized the bending energy to describe the elasticity of lipid membranes, proposing a free energy, which depends on a bending rigidity modulus κ. For a bilayer, the bending modulus depends on the area-compression modulus $K_{A}$, which represents the energetic cost of expand/compress the area of the a single layer. Hence, assuming a homogeneous layer and considering a pure bending deformation, the general elastic energy reduces to the bending contribution.

### 3.1. Cell Membrane Dynamics

In recent years, several numerical models to understand and replicate the elastic properties of cells have been developed [34]. Various numerical techniques have been reported to model a single RBC’s mechanics and its elastic properties, such as the finite element method [35], boundary integral models [36,37] lattice-Boltzmann method [38,39,40] and dissipative particle dynamics [41,42]. Most of these methods use a multiscale approach for single-cell modeling.

The representation of the membrane as a two-dimensional layer is reasonably accurate, the simplest and most direct formulation consists of defining a mesh of points which represents the membrane neutral surface, and from there extract the local mean curvature or deformation tensor necessary to compute the elastic energy. The most important examples include the immersed boundary methods [43,44], integral boundary methods [45,46] or multiparticle collision dynamics [47,48]. Methods in this direction have been successfully applied to the study of many membrane-related topics [31,49]. All these methods require of an explicit tracking of the membrane position and the calculation of the deformation variables, i.e., the curvature.

A different approach, based on a Eulerian rather than a Lagrangian description, are the phase-field models [50]. The membrane is identified from an auxiliary scalar field defined in the entire space, and the method details the dynamics of the field, instead of specifically dealing with the evolution of the interface. This formulation also avoids the problem of defining the boundary conditions at the membrane surface. Although the application of phase-field methods to amphiphilic systems was extensively investigated in the past [51], it is only recently that these models have been used in the study of cell morphology and dynamic response [39,52,53,54].

Combining the Helfrich free energy model, mentioned earlier in this section, and a phase field method, the dynamics of a membrane are defined as a function of an order parameter ϕ, which varies between −1 and 1, defined as $\Phi \left[\phi \right]=-\phi +\phi^{3}-\epsilon^{2}\nabla^{2}\phi$ and a mean bending modulus $\bar{\kappa }=\frac{3\sqrt{2}}{4\epsilon^{3}}\kappa$. Here, ϵ is the interfacial width, and the order parameter is given as $\phi \left(x\right)=tanh(x/\left(\sqrt{2}\epsilon \right))$ [55]. The dynamic of the membrane is described as

(1) $$\frac{\partial \phi }{\partial t}=\bar{\kappa }\nabla^{2}\left\{\left(3\phi^{2}-1\right)\Phi \left[\phi \right]-\epsilon^{2}\nabla^{2}\Phi \left[\phi \right]+\epsilon^{2}\bar{\sigma }\left(x\right)\nabla^{2}\phi +\epsilon^{2}\nabla \bar{\sigma }\left(x\right)\cdot \nabla \phi \right\},$$

where the term $\mu_{mem}=\left(3\phi^{2}-1\right)\Phi \left[\phi \right]-\epsilon^{2}\nabla^{2}\Phi \left[\phi \right]+\epsilon^{2}\bar{\sigma }\left(x\right)\nabla^{2}\phi$ represents the chemical potential of the membrane. The parameter σ is the mean surface tension of the membrane defined as $\bar{\sigma }\left(x\right)=\frac{\sqrt{2}}{6\epsilon^{3}\bar{\kappa }}\sigma \left(x\right)$.

### 3.2. Membrane Dynamics and Hydrodynamic Coupling

The dynamics of the membrane are dictated by Equation (1), but, in many systems, the hydrodynamic effects of the aqueous environment are also crucial in the membrane evolution. A typical example is the study of lipid vesicles in shear flow [56,57], which serves as a model system for RBCs while flowing along capillaries forced by an external flow. To model the interaction of the membrane with the surrounding fluid, the Navier–Stokes equation is frequently used to describe the dynamics of the fluid, and both equations are coupled describing the membrane–fluid interaction. The complete Navier–Stokes phase-field model (NS-PF) [58,59] is

(2) $$\frac{\partial \phi }{\partial t}+v\cdot \nabla \phi =M\nabla^{2}\mu_{mem},$$

(3) $$\rho \left[\frac{\partial v}{\partial t}+\left(v\cdot \nabla v\right)\right]=-\nabla P-\phi \nabla \mu_{mem}+\eta \nabla^{2}v+f_{\mathrm{ext}},$$

where ϕ is the order parameter, v is the velocity of the fluid, $\mu_{mem}$ is the chemical potential of the membrane, ρ is the density of the suspension, *P* is the pressure exerted on the fluid and $f_{ext}$ are the external forces applied to the fluid.

From the perspective of RBC elasticity, the membrane mechanics are often characterized with the bending and shear modulii. The minimization of the Helfrich free energy for an ellipsoidic shape under the appropriate values of area and volume leads to the biconcave discocyte of the RBC as the equilibrium shape. Nevertheless, to obtain an accurate model of RBC membranes, the cytoskeleton’s elastic properties must be considered. The cytoskeleton presents a low resistance to bend, with a bending modulus at least two orders of magnitude lower than that of the bilayer. It does present, however, resistance to shear and compression in the membrane layer, and it is known to play a fundamental role in inhibiting budding and vesiculation processes. Different models have been formulated to model the cytoskeleton’s elasticity. A simple way is to represent it as a spring mesh, relating the spring constant with the elastic modulii. A different approach is to recover the continuum mechanics description and consider the finite strain theory [60].

The elastic properties of the RBC membrane are highly dependent on the specific bilayer lipid composition, ATP concentration, age of the cell, and temperature. They are also known to vary with the morphological state of the cell, and echinocytes or spherocytes are considerably more rigid than discocytes. The bending rigidity has been measured by different experimental techniques [61], such as, micropipette [1], AFM [62] and optical tweezers [63]. Typical values fall between 10 and 50$\;k_{B}T$, with slight deviations depending on the specific technique. The shear modulus of the bilayer is negligible due to its fluidic nature in the membrane plane, given that any shear stress is instantaneously relaxed by the rapid lateral rearrangement of lipids.

With the improvement of miniaturization techniques and methods in the past 20 years, microfluidics has become a fundamental aspect for studying the elastic and mechanical properties of the RBCs from an experimental point of view. As a result, several studies have been successful in relating theoretical and numerical analyses with experiments [7,64,65].

## 4. Human Red Blood Cells and Blood Components

### 4.1. Human Red Blood Cells

**Figure 2:**
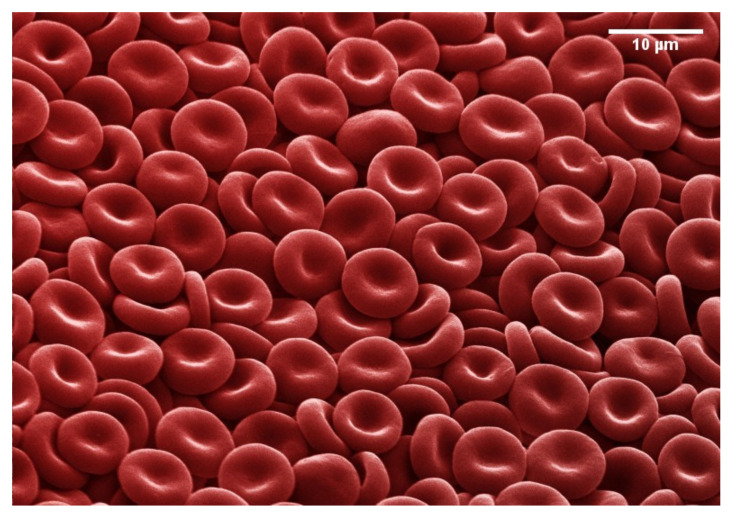
Photograph of RBCs. In this picture, the biconcave disc shape of RBCs is observed. The RBCs are from a healthy 18-year-old male and were imaged on a SEM microscope as quickly as possible so the blood cells did not shrink and distort. The image has been digitally modified to add the red color typical of RBCs. Credits: Annie Cavanagh available under Creative Commons by-nc-nd 4.0 from http://wellcomeimages.org/, accessed on 30 January 2020.

Mammalian RBCs have different shapes and sizes, depending on the animal’s physiological requirements (e.g., oxygen consumption in animals inhabiting high-altitude mountains). Human RBCs have a disk shape with a typical diameter of 8 μm, with a concave region in the center where the cell achieves its minimum thickness of 1 μm, and a convex outer rim where it reaches a maximum thickness of 2 μm; see Figure 2. This particular shape is usually known as the biconcave discocyte, and it corresponds to the healthy state of the cell. The typical cell area and volume of a healthy individual are 140 μ$m^{2}$ and 90 μ$m^{3}$ [66], respectively. Cells present specific regulatory systems to maintain their area and volume constant, thus ensuring that their resting shape is fixed.

In humans, RBCs exhibit a huge intraindividual variability, with strong correlation with sex and age. Cells of men are up to 20% larger than in women, and men also present higher hematocrit in the circulatory system. Aging affects to the RBC membrane rigidity, so that old individuals present more rigid cells. The biconcave discocyte, however, represents just one of the many morphologies exhibited by RBCs, and it responds to a very specific conditions of area-to-volume ratio, bilayer and cytoskeleton elastic properties, membrane internal asymmetry and pH of the surrounding aqueous environment, among others. Other well-known morphologies are the stomatocyte, when the cell acquires a cup-like shape, and the echynocyte, when the cell becomes spherical and it develops many spikes around its contour. The entire shape deformation comprises a sequence of different morphologies usually known as stomato-discoechynocyte [67], and it is triggered by the disruption of the membrane microstructure which changes the membrane asymmetry.

The origins of the peculiar discocyte shape have been subject to debate for decades. It seems reasonable that the large cell area compared to volume (compared to that of a sphere, the so-called reduced volume $v_{red}=V/\left(4\pi R^{2}/3\right)=0.6$, where $R=\sqrt{A/(4\pi )}$ is the radius of a sphere with equal area to the cell; thus, for a sphere, $v_{red}=1$), responds to the necessity of optimizing the diffusion of oxygen across the membrane. Alternatively, it has been proposed that the disk has a low inertial momentum, so that it does not rotate when flowing in the main arteries, minimizing the formation of turbulent flows [68]. Another hypothesis postulates that the discocyte is an appropriate shape to undergo strong deformations and pass through the smallest capillaries, after recovering the normal relaxed shape [69].

Three fundamental effects, derived from the characteristic geometry of RBCs, affect blood flow: (1) The geometry gives them the capacity to align with the direction of flow. (2) The cellular membrane of a healthy RBC is flexible, which means that it can change its shape and deform under different flow conditions. (3) RBCs’ shape facilitates their adhesion together, forming aggregates. All these properties of the RBCs act together to give blood a viscosity that is substantially higher than blood plasma and contribute to its non-Newtonian properties.

### 4.2. Blood Components

Human blood is a two-phase fluid system and consists mainly of an aqueous polymeric and ionic solution of low viscosity, the plasma, in which is suspended a 0.45–0.50 concentrated cellular fraction [70]. The plasma is a liquid-phase mixture of metabolites, proteins and lipoproteins suspended in a salt solution composed mostly of water. The cellular fraction is a complex mixture of erythrocytes (RBCs), leukocytes (white blood cells), and thrombocytes (platelets). Nearly 99% of the cellular fraction in blood is represented by RBCs [71]. The complete set of blood components is usually referred to as whole blood. Given the complex constitution of blood, it is considered as a non-Newtonian fluids, which presents a shear-thinning behavior. The rheological properties of blood are primarily due to the diversity and particular features of its constituents.

Human blood plasma is known to behave as a Newtonian fluid; however, some recent studies have observed viscoelastic behavior in human blood plasma [72]. Plasma proteins play an important role in the hemorheological properties of whole blood. First, even though blood plasma is ≈92% water, due to plasma proteins, its viscosity at $37{\;}^{\circ }$C is around 1.7 times the viscosity of water at the same temperature [73]. Second, plasma proteins (especially fibrinogen) cause RBCs to stick together, forming aggregates, such as piles of coins, known as rouleaux. Rouleaux formation is important because it causes the viscosity of blood to be very dependent on the shear rate to which it is exposed [74].

The erythrocytes’ volume fraction in blood is commonly referred as hematocrit. The normal range of hematocrit differs between men and women, 40 to 50% and 36 to 46%, respectively. Leukocytes and thrombocytes together only comprise about 1% of the cellular fraction. This high concentration of RBCs is the main reason that they are hemorheologically important. Additionally, the physical and morphological properties of RBCs also contribute to the non-Newtonian behavior of blood.

White blood cells (WBCs) and platelets do not have a significant hemorheological role, mainly due to their low concentration in blood in comparison with RBCs. Despite WBCs being bigger in size, presenting viscoelastic properties by themselves, and playing an important role in microcirculation resistance, their volume concentration is approximately three orders of magnitude lower than RBCs. Thus, their effects are less relevant in general circulation. In the case of platelets, they are much smaller than RBCs (2–4 μm) and their volume in blood is even smaller than the leukocytes’ volume. As a consequence, they neither influence whole-blood viscosity directly nor microvascular resistance. However, recent studies have considered the biomechanics of platelets to be fundamental in clinical diagnostics [75,76].

## 5. Hemodynamics and Hemorheology

The circulatory system is an organ system that circulates blood along all the cells and tissues, facilitating the transport of oxygen and nutrients, which allows the nourishment of the cells [77]. It also serves as a carrier of other molecules or matter, and is used in processes such as the transport of waste products towards the excretory system, or a fast transport of hormones from one part of the body to another in response to a certain environmental condition [78]. Generally, the main function of the circulatory system is to provide the molecules that the body tissues need at each moment.

The circulatory circuit is composed of a collection of blood vessels. These blood vessels decrease in size from the arteries and veins, through arterioles and venules, to capillaries where they reach the organ tissues and nutrient exchange takes place. The circulation of blood in these microvessels: arterioles, venules and capillaries is know as microcirculation [79].

Blood circulates constantly around the body, and therefore the study of blood flow and its rheological properties is crucial to understand the processes underlying microcirculation. Moreover, several studies have demonstrated that the alteration of hemodynamics and hemorheology is associated with various diseases that affect the normal circulation of blood [16,80,81,82]. In this section, we will review the general features of hemodynamics and hemorheology, and the contribution of recent numerical and experimental microfluidic techniques, to study the hemodynamical and hemorheological properties of blood from a single-cell effect to their collective behavior.

### 5.1. Hemodynamics and Hemorheology for a Single Cell

Hemodynamics is the area of biophysics and physiology that studies the fluid dynamics of blood flow inside the different structures of the circulatory system: arteries, veins, arterioles, venules and capillaries. Blood flow in the human body is affected by several factors, such as the driving pressure of the flow, the flow characteristics of blood and the geometric structure and mechanical properties of blood vessels [83]. Hemodynamics research has a long history and is an attractive topic, with several theoretical, experimental and computational studies having been developed in the past 50 years [84,85,86,87,88]. The field continues to expand due to recent advancements in numerical and experimental techniques at the microscale. These new techniques have enabled the prediction and observation of blood flow in vitro, emulating in vivo conditions. The combination of computational hydrodynamics and microfluidics have become key elements to approximating the blood flow in the microcirculatory system.

Blood circulates the human body pumped by the heart, which generates a pressure difference in the system. As the blood flows through the circulatory system, the pressure falls progressively by the time it reaches the termination of the venae cavae where they empty into the right atrium of the heart [78]. The heart pumping is pulsatile, and therefore the arterial pressure alternates between a systolic pressure level and a diastolic pressure level. This pressure difference allows blood to flow through the different blood vessels in our body, enabling microcirculation.

Microcirculation flow is characterized by a low Reynolds number $Re=\frac{\eta vD}{\rho }<1$, where *v* is the velocity of the flow, *D* is the diameter of the microvessel, η is the dynamic viscosity of the fluid and ρ is the fluid density. The Reynolds number is defined as the ratio between the inertial and viscous forces, and in the microvessels it ranges between 0.001<Re<0.1. Hence, the viscous effects are more significant than the inertial effects, and the flow is laminar. In microvessels over 200 μm diameter, it can be assumed that blood is a homogeneous continuous fluid. This assumption is not true for smaller microvessels and here the individual motion of RBCs becomes important. When small objects, such as droplets or cell, enter a microchannel, the hydraulic resistance along the channel is given as the addition of the resistance of the channel in the absence of the particle and the resistance developed across the length of the object. The resistance of the object will depend on the local characteristics of the flow and the viscoelastic properties of the object [89].

To measure the factors that affect hemodynamics, several numerical and experimental techniques are used, such as dielectrophoresis, magnetic interaction, optical traps and biomarkers [14]. Using these techniques, researchers have been able to study blood flow behavior from a single RBC to their collective behavior and blood as a homogeneous fluid. When studying blood flow in confined geometries for a single cells, the effect of the system walls are relevant, enabling RBCs to form a single train at the center of the microchannel. However, when studying the collective behavior of RBCs, a focusing phenomenon arise due to the presence of walls and cells to cells interactions. The effects of focusing, or RBC migration, affects the rheological properties of blood, affecting its viscosity and therefore the blood flow.

From a numerical point of view, several techniques have been reported to model RBCs in blood flow [90,91,92,93,94]. Most of these methods have in common a multiscale approach for single-cell modeling in confined geometries. Confinement is crucial to exploring the RBCs’ elasticity effect in microcirculation, since it replicates the in vivo conditions of blood circulation.

To model the interaction between RBC membrane elasticity and flow, a dimensionless quantity, $C_{\kappa }$ (usually referred to as capillary number) is defined as the ratio between the elastic relaxation time, $\tau_{m}$, of the membrane of cells suspended in the fluid, and a viscous time $\tau_{\eta }$ associated with the viscous forces of the fluid [58,59]

(4) $$C_{\kappa }=\frac{\tau_{\kappa }}{\tau_{\eta }}=\frac{\eta_{0}{\bar{v}}_{z}d^{2}}{\kappa }\left(\frac{d}{b}\right),$$

where ${\bar{v}}_{z}$ is the mean velocity in the direction of the flow, $\eta_{0}$ is the viscosity of the surrounding fluid, *d* is the diameter of the RBC d≈ 8 μm, *b* is a geometrical parameter which accounts for the confinement and κ is the bending modulus. For human RBCs, the typical values of the bending modulus are $\kappa \approx 50k_{B}T=2\times 10^{-19}$ J. The ratio d/b is a parameter that relates the RBC size to the size of the system to establish the confinement. The dynamics of the cell membrane are controlled by the viscosity ratio between the inner and outer regions of the cell, and the capillary number which characterizes the shear rate of the force relative to the membrane rigidity. The effective (or apparent) viscosity of the whole suspension (i.e., liquid and cells) is computed from the relation of the applied force, $f_{0}$, and the outcome flow given by the mean velocity ${\bar{v}}_{z}$,

(5) $$\eta_{eff}=\frac{f_{0}}{12{\bar{v}}_{z}}b^{2}.$$

**Figure 3:**
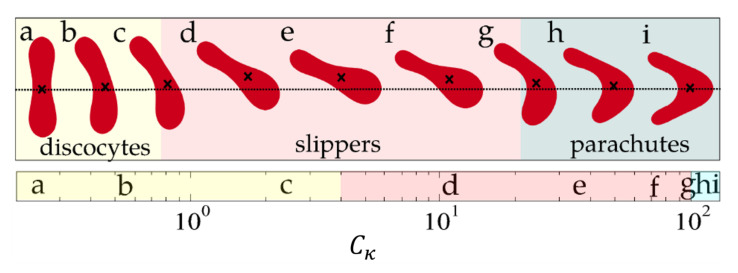
Red blood cell morphologies in a Poiseuille flow, modelled through Equations (4) and (5) for an increasing capillary number, $C_{\kappa }$. The letters associated to the red blood cells represent different stages of the cell morphology. The parameter used to model the RBC was a reduced volume $v_{red}=0.48$ and a confinement d/b=0.71, defined as the ratio between the RBC size and the system size. The colored regions represent the three main morphological regimes, namely the discocyte (yellow), the slipper (red), and the parachute (blue). The dotted line represents the channel axis, and the crosses are the center of mass of each RBC. Image reproduced from Lázaro et al. (2014) [58].

**Figure 4:**
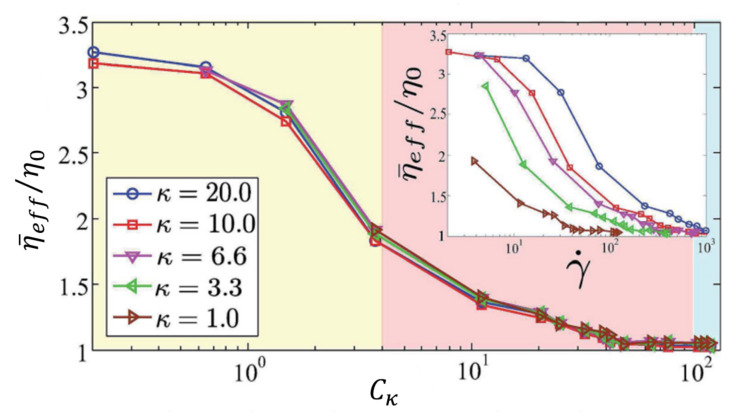
Effective viscosity of an RBC suspension as a function of the capillary number for different bending rigidities, κ, at confinement is d/b≈0.71. The value of $\eta_{eff}$, obtained from Equation (5), is averaged for different initial conditions of the RBC. The coloured regions correspond to the three morphological regimes shown in Figure 3. The curves for different rigidities as a function of the shear rate show the sensitivity of the viscosity and RBC morphology to the rigidity of its membrane; however, the curves collapse when the relative effect between the viscous and elastic forces is considered. Image reproduced from Lázaro et al. (2014) [58].

Figure 3 shows three different RBC morphologies in a Poiseuille flow, modeled using the Navier–Stokes phase field models discussed in Section 3, as a function of the capillary number [58,59]. The effects of the hydrodynamics on the viscosity of the solution is shown in Figure 4.

From an experimental point of view, early hemodynamical experiments only provided a qualitative understanding of blood flow. Quantitative information, such as rheological effects and blood cell deformability were difficult to obtain due to lack of time and spatial resolution. Eventually, high-speed and high-resolution cameras, with an enhanced sensitivity and mounted to an optical microscope, enabled velocity measurements of such small-scale flows. In this aspect, several techniques have been developed to measure the velocity fields of blood and RBCs at the microscale, such as μPIV (microparticles image velocimetry) or PTV (particle tracking velocimetry) and wavelet-based optical flow velocimetry (wOFV) [95,96,97,98,99].

**Figure 5:**
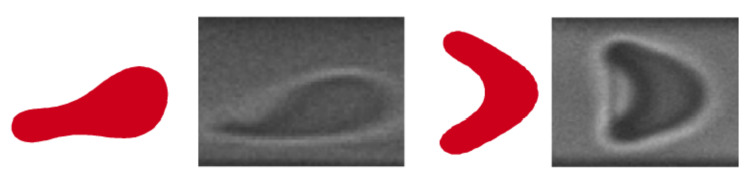
Comparison between slipper (**left**) and parachutes (**right**) obtained from numerical and experimental results. Numerical images reproduced from G. R. Lazaro et al. (2014) [58]. Experimental snapshots adapted by permission from RSC, G. Tomaiuolo et al. (2009) [7] under license 1181911-1.

The rise of microfluidics in the last two decades has enabled the increase in experimental options to study RBCs’ properties and their effect on blood flow [89,100,101,102]. The easiness of replicating small structures in microfluidics allowed the development of various designs and structures to observe and analyze the deformation of RBCs [103,104,105]. Typical microfluidics approaches consider a forced, or gradual, constriction of RBCs, as they circulate through very narrow slits. The viscoelastic properties of the RBC membrane are obtained, establishing a relation between the shear flow and the pressure gradient applied to them [106]. Moreover, the combination of numerical and experimental models has enabled a deeper study of the biomechanical properties of RBCs [107,108] and their sensitivity to blood flow [109]. These have been successful in capturing several changes in the morphology of the RBCs, replicating the slipper and parachute shapes of RBCs under shear flow [65,110]; see Figure 5. Other studies on the elasticity of RBCs have submitted them to extreme deformation circulating through submicrons slits [111], to simulate the filtration of RBCs in the spleen.

### 5.2. Experimental Hemorheology: Collective Behavior of Red Blood Cells

Hemorheology is the study of the rheological properties that affect blood flow. These properties are mostly related to the non-Newtonian nature of blood, due to its composition and to the biomechanics of its erythrocytes. In vivo, blood flow is determined by multiple factors, including hematocrit levels, RBCs deformability, the elasticity of venules and arteries, and blood pressure. The rheological properties of blood have been studied for many years and it has been demonstrated that it presents a shear-thinning behavior [112,113,114,115], which means that, as the flow velocity increases, the viscosity of blood decreases. This rheological properties of blood highly depend on the properties of its RBCs, which affect the viscosity of blood, as well as its shear-thinning behavior. Typical values for the viscosity of healthy blood at a low shear rate (0.28 s${}^{-1}$) are 39±4 mPas for females and 48±6 mPas for males. At high shear rates (128 s${-}^{1}$), the viscosity values are 4.3±0.2 mPas and 4.7±0.2 mPas for females and males, respectively [116].

From a macrorheological point of view, the viscosity of blood is directly related to the fraction of RBCs suspended in plasma. Given the particular properties of RBCs, the increase or decrease in its concentration in plasma (hematocrit) will affect the behavior of blood, where the erythrocyte concentration is directly proportional to the viscosity [117]. Therefore, increased hematocrit levels will lead to an increased viscosity of blood and decreased hematocrit levels will lead to a decreased viscosity. Thus, the concentration of RBCs in plasma affects the whole-blood viscosity values, as well as its non-Newtonian behavior, which is lost at low hematocrit levels [118]. From a microscopical point of view, two properties of RBCs are particularly important in the effort to understand the shear-thinning behavior of blood: their deformability [119] and their tendency to form aggregates [74,120,121]. Both properties cause the highly non-Newtonian behavior observed for RBC suspensions in plasma [122]. At low shear rates, the viscosity of blood is high, whereas, at a high shear rate, red cell disaggregation and deformation reduces the viscosity of blood.

**Figure 6:**
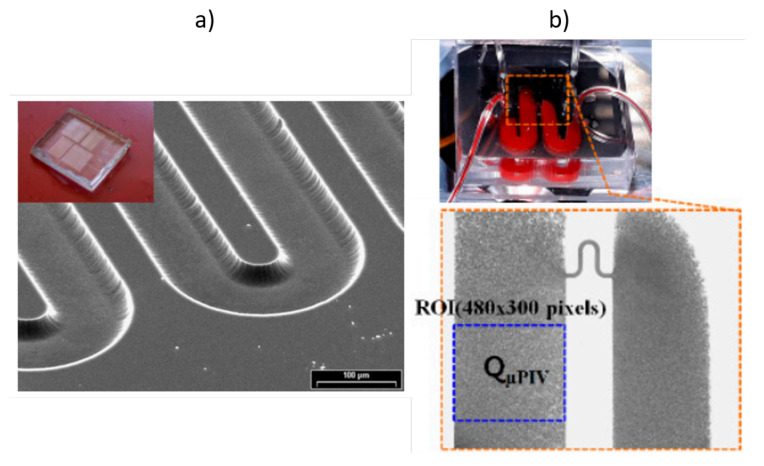
Images of two different microfluidics devices developed to measure blood viscosity. (**a**) Image reprinted by permission from Springer Nature, Morhell and Pastoriza (2013) [135] under license 5235930238113. (**b**) Image adapted by permission from MDPI Kang (2018) [132] under Creative Commons CC by 4.0 license.

The viscosity of fluids is measured using viscometers. Complex fluids’, such as blood, rheological properties are studied using a rheometer, capable of measuring their behavior under different flow conditions. Rheometers differentiate from the type of flow they induce on a material; these may be drag or pressure-induced flows. Typical drag flow rheometers are cone-plate and cylindrical Couette rheometers. On the other hand, the most typical pressure-driven flow is the capillary rheometer. The capillary rheometer was the first rheometer, and is still the most common method to measure viscosity, due its low cost and easy operation. In comparison with rotational rheometers (cone-plate and Couette), they can be closed devices, which avoid the evaporation of solvents and the expulsion of samples. Capillary viscometers and rheometers have been used since the beginning of hemorheology in the 1960s, to measure the viscosity of blood plasma and blood [123]. However, the rise of microfluidics at the end of the 1990s brought new applications and innovation in this area. In recent years, a variety of microfluidics devices and methods have been developed with the objective of measuring the viscosity of blood plasma [124,125,126,127] and blood [128], using optical detection techniques [75,129,130,131,132,133,134], pressure sensors [135,136] and electrical sensors [137,138,139]; see Figure 6.

In a capillary, the viscosity of the fluid is measured by establishing the relation between the pressure difference exerted on the fluid and the flow velocity. The pressure difference, which moves the fluid inside the microchannel, is generated through gravity, gas compression, pistons or suction. For Newtonian fluids, the viscosity is determined as the ratio between the shear stress, σ, defined as a function of the pressure exerted on the fluid, and the shear rate, $\dot{\gamma }$, defined as a function of the velocity. However, for non-Newtonian fluids, the relation between these parameter becomes non-linear and the viscosity of blood is measured through a local relation between the shear stress and the shear rate. Typical non-Newtonian viscosity models used for blood are the power law, the Carreau, the Carreau–Yasuda and the Casson models [140]. Nonetheless, the simplicity of the two parameters of the power law model makes it the most popular model used to estimate blood viscosity. This model states that the viscosity of the fluid is defined as a function of the shear rate through:

(6) $$\eta =m{\dot{\gamma }}^{n-1},$$

where *m* is a consistency factor that depends on the fluid and *n* is the behavior factor that defines the character of the fluid. When n=1 the fluid is Newtonian, for n<1 the fluid is shear thinning and for n>1 the fluids is shear thickening.

**Figure 7:**
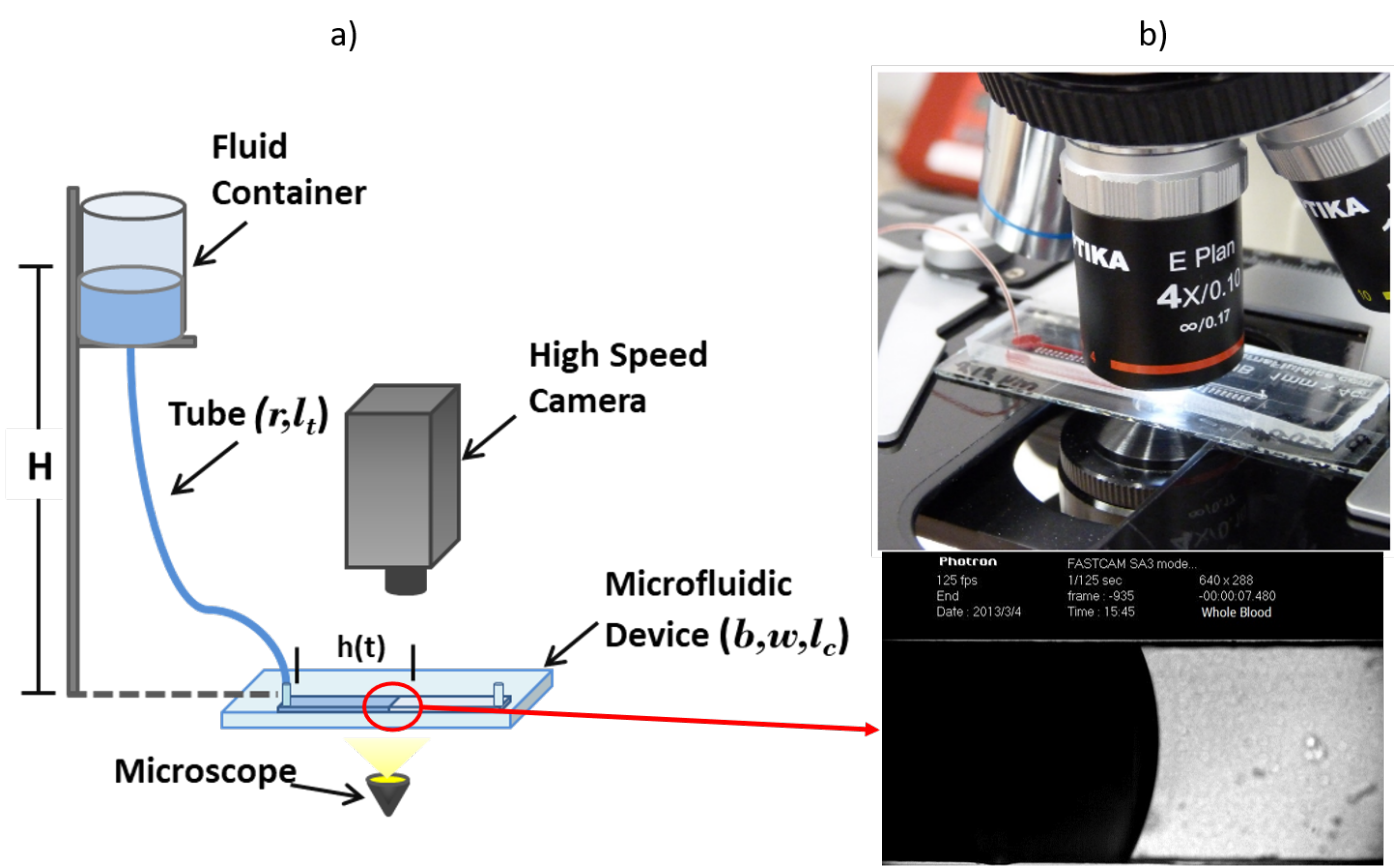
(**a**) Schematic representation of the experimental set up to perform blood viscosity measurements using microfluidics. The pressure difference is generated through hydrostatic pressure $P_{hyd}=\rho gH$, where *H* is the height from the fluid in the reservoir to a microchannel of width *w*, depth *b* and length $l_{c}$. These are connected through a tube of radius *r* and length $l_{t}$. (**b**) Photograph of the experimental set up. Top image: A view of the microdevice under a microscope. Bottom image: View of the blood–air interface inside the microfluidic channel, taken with an Optika XDS-3 microscope and a high-speed camera Photron Fastcam Viewer 3. Images reproduced from the work of Trejo-Soto et al. (2017) [130].

A basic method to determine the viscosity of blood is the Front Microrheology method, which consists of inducing a pressure difference in the fluid through hydrostatic pressure $P_{hyd}=\rho gH$. The pressure is controlled through a fluid column inside a reservoir set at different heights *H* and connected to a bio-compatible tube with uniform internal cross-sections of radius *r* and length $l_{t}$. The tube connects the reservoir with a rectangular microchannel of width w=1 mm, depth b=0.3 mm and length $l_{c}=4$ cm, fabricated in PDMS over a glass substrate using typical microfabrication techniques [141,142,143]. Figure 7a shows a schematic view of the experimental setup described. The observation of the blood–air interface (blood front) inside the microchannel is made using a microscope and a high-speed camera; see Figure 7b. The velocity of the blood front is measured tracking the mean front position as a function of time between several contiguous images. A full description of the microfluidic device and details of the experimental method are reported by Trejo-Soto et al. (2016) [127].

**Figure 8:**
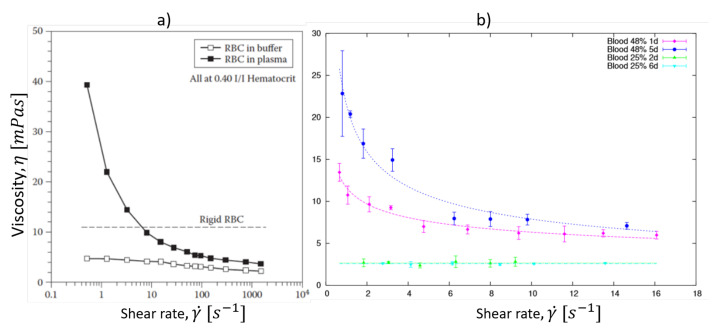
Viscosity of blood as function of the shear rate. In both images, the non-Newtonian nature of whole blood is shown. At a low shear rate, RBCs form aggregates which are responsible for an increase in viscosity. As the shear rate increases, cells disaggregate and move freely through blood vessels. If the shear rate keep increasing, RBCs deform, elongate and align with the direction of the flow, which happen in microcapillary vessels. The image on the **left** (**a**) shows one of the first viscosity measures of blood obtained using a typical rheometer, image reproduced from Baskurt et al. (2007) [116]. On the **right** (**b**), shows the viscosity of a fresh 48% hematocrit blood sample (magenta) and the same sample 5 days from extraction (blue), we observe how the aging of the sample affects the viscosity of the sample. Image reproduced from Trejo-Soto et al. (2017) [130].

According to this experimental setup, an effective pressure $\Delta P_{eff}=\rho gH-P_{L}$ is defined, where $P_{L}$ is the Laplace pressure due to the curvature of the fluid interface. This effective pressure is related to the stress through [130]:

(7) $$\sigma =\frac{r}{2l_{t}}\left(\rho gH-P_{L}\right),$$

where *r* and $l_{t}$ are the internal radius and the length of the tube, respectively. The shear rate of the system is defined as a function of the velocity of the interface, and the geometrical parameter of the coupled system tube-microchannel, through the following expression:

(8) $${\dot{\gamma }}_{F}\left(n\right)=\frac{b^{2}w}{\pi r^{3}}\left(3+\frac{1}{n}\right)\frac{v}{b},$$

where *b* and *w* are the geometrical parameters of the experimental microchannel. The parameter *n* is the behavior exponent obtained using a power law model to describe the viscosity of blood. The viscosity of blood and its shear-thinning behavior have been measured and observed using several methods [144]; see Figure 8. Using the power law model, typical values of the exponent for blood are around n=0.80 [129,136].

Although this standard procedure provides important information about the bulk behavior of the fluid, it is of limited interest for understanding the flow in very confined systems, when the rheological behavior can be severely affected. For a single cell, elastic properties are more relevant, and RBCs as an ensemble are mainly affected by confinement and focusing.

### 5.3. Comparison with Numerical Results of the Collective Behavior of RBCs

As mentioned earlier, the flow of RBCs in tubes and channels is critically controlled by the hematocrit. The interactions between the RBCs, involving hydrodynamic interactions, purely geometrical constraints, or aggregation, play a fundamental role in the collective dynamics of the RBC suspension. At low concentrations, vesicles and hard spheres flowing in thick tubes migrate from the center line and reach a stable trajectory at ≈0.6r from the axis, forming an annulus of high density at this radial distance, the so-called Segre–Silberberg effect [145]. At high concentrations, however, RBCs distribute along the tube core, avoiding the region close to the wall. The transition from the single-cell to the high hematocrit behavior is still poorly understood in spite of its importance in the rheological behavior of the fluid.

When blood measures are at the microscale, other effects may be observed, namely, the Fåhraeus [146] and the Fåhraeus–Lindqvist effects [147]. The latter, characterized by a dependence of the blood viscosity with the channel thickness, are perhaps the most important example [148]. In the range between roughly 300μm and 10μm of the tube diameter, the effective viscosity decreases up to 4–5 times. This effect occurs as a consequence of the strong repulsion from the walls that forces the blood cells to concentrate on the central region of the channel. The formation of layers free of cells close to the walls allows a rapid flow in these regions, enhancing the overall fluidity. At high confinements, the walls’ proximity enforces a more concentrated distribution of cells in the center and consequently broader layers of free flow are present [149]. In larger channels, the free layers are proportionally thinner until their effect becomes eventually negligible. Although, in the narrowest channels (<10μm), RBCs are ordered in a single row for flow at low concentrations, and thus interactions between cells are disregardable. At an intermediate channel size (≈20μm), RBCs present a more complex behavior and collective effects must be considered [150].

While flowing in thicker channels, where cells typically flow at higher concentrations, RBCs do interact, and collective effects substantially change the flow properties. From a theoretical point of view, the organization in trains (observed for single cells) also offers an interesting way to study the hydrodynamic interactions between neighboring cells, and how it affects the RBCs’ dynamics. The membrane stiffness dictates the flow disruption induced by the RBCs. Rigid cells induce stronger perturbations of the incoming flow than softer ones. Even if deviations from the imposed flow are small when RBCs are distant, interactions strengthen for lower distances between cells, favoring RBCs’ collective behavior. If RBCs are initially placed very close to each other, even at high capillary numbers, they do not migrate towards the walls but flow whilst maintaining a centered position.

**Figure 9:**
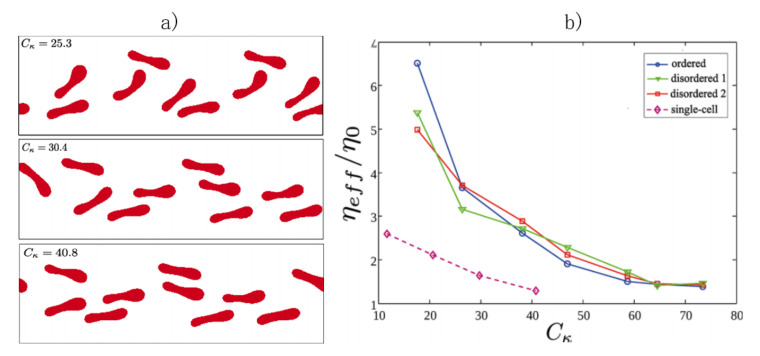
(**a**) Simulation of RBCs during flow in a confined channel. (**b**) Numerical results of the effective viscosity for an RBC suspension at a low concentration of RBCs and low confinement as a function of the capillary number, Equation (5). Three initial conditions, one ordered and two disordered, are calculated, obtaining similar results. The curve shows the expected shear-thinning behavior, and the differences with the single-cell case. Image reproduced from Lázaro et al. (2019) [151].

RBCs are very sensitive to the hydrodynamic interactions with other cells, and the competition between these interactions and the wall effects dictates different RBC flow properties when several cells are flowing at high and moderate concentrations. For instance, in the inertial regime, the limit of single-cell behavior is characterized by the Segré–Silberberg effect, when cells migrate towards a specific lateral position, whereas at higher concentrations the collective behavior dominates and cells are located at the tube core, the Fåhraeus–Lindqvist effect. The dynamics of several RBCs at moderate concentrations have proven to differ in several aspects from the single-cell case, and this affects the rheological behavior of the suspension. Recent numerical analyses have computed the effective viscosity for three configurations (one ordered and two disordered initial conditions), at a low volume fraction and concluded that the viscosity curves show the expected shear-thinning behavior, though two main differences were found with respect to the single-cell case: the magnitude of the effective viscosity obtained and the $C_{\kappa }$, Equation (4), required to observe the shear-thinning decay [151]; see Figure 9.

**Figure 10:**
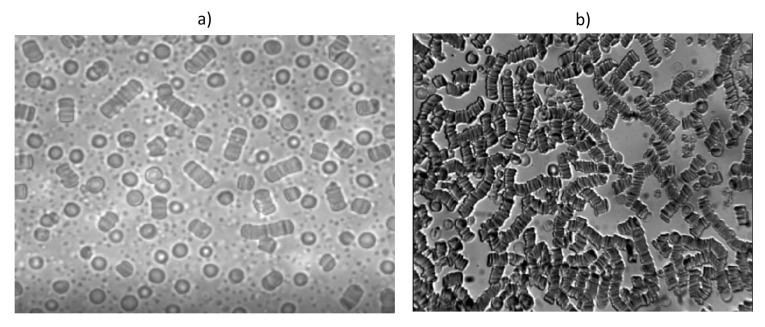
(**a**) Red blood cells aggregates from a blood sample at 5% hematocrit. The image was taken with an inverted Optika XDS-3 microscope using a 50× magnitude objective. (**b**) Large RBC aggregate from a blood sample at 38% hematocrit. The image was taken with an Optika B-353LDI microscope using a 40× magnitude objective.

Other important characteristics of RBCs in their collective interaction is their aggregation. RBCs have a tendency to form stacked structures (aggregation), commonly known as rouleaux. The formation of aggregates affects blood flow and its rheological properties, increasing blood viscosity, and therefore slowing down the flow. These structures have several characteristics, such as, the number of RBCs per rouleau being variable and side-to-side formations being possible, due to the particular discocyte shape of the RBCs [74]. Figure 10 shows two images of rouleaux formation of two blood samples with different hematocrit levels, where some of these characteristics are observed.

**Figure 11:**
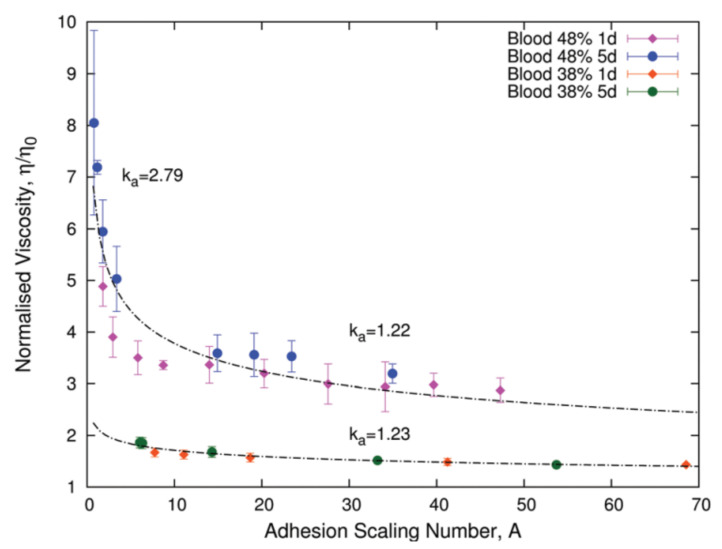
The plot shows how the normalized viscosity, $\eta /\eta_{0}$, follows a universal curve as the adhesion scaling number changes. Image reproduced from Trejo-Soto et al. (2017) [130].

Aggregation has rheological consequences in blood and it determines its non- Newtonian behavior at low shear rates. When RBCs are aggregated, more shear is required to move the fluid, but as shear increases, RBCs start to disaggregate, making it easier to change the state of motion of blood. If we keep increasing the shear, cells start to align with the flow, deform, and elongate. Therefore, if aggregation increases, then blood viscosity increases as well and the shear-thinning behavior of blood is altered. Numerical studies have demonstrated the effects of aggregation in blood viscosity [120,122,152,153,154] and experimental studies have observed that RBCs’ deformability induces cell aggregation during flow in microcapillaries, allowing the formation of clusters of cells [98,155,156]. Moreover, the aging of storaged RBCs also contributes to the increase in aggregation and affects its viscosity. However, when scaling according to adhesion energies, a collapse in the viscosity curves defines a single universal behavior for blood viscosity [130]. To analyze the effects of aging, the hematocrit levels were fixed and the behavior of the blood sample as it ages was studied, showing that as the sample aged, the aggregate formation increased. By introducing a non-linear scaling parameter, the adhesion scaling number, A, the effects of aging on RBC aggregation was quantified. This quantity is defined as

(9) $$A=\frac{\eta_{0}\dot{\gamma }d^{3}}{k_{a}E_{0}},$$

where $\eta_{0}$ is the viscosity of plasma, *d* is the average diameter of an RBC, $E_{0}$ is the energy scale associated with the aggregation energy between RBCs [122], and $k_{a}$ is a scaling factor that accounts for the relative increase in the adhesion energy when the blood ages. Then, the parameter *A* can be interpreted as the ratio between the characteristic viscous energy scale and the aggregation energy. When the viscosity of the fluid is normalized according to the hematocrit levels and blood plasma $\eta_{eff}=\eta /\eta_{0}$, the changes in the viscosity depend only on the adhesion scaling number. Figure 11a shows the effect on blood viscosity due to RBC aggregation, induced by aging.

In general, blood rheology has been proven to be experimentally difficult to measure. Thus, inaccurate interpretations have frequently been made [157]. Many microfluidics techniques have been able to reduce difficulties using capillary rheometry and pressure-driven flow. The advantages of microfluidics in this area stand out, mainly portability and the need for only a small sample. Additionally, some microfluidic techniques related experimentally blood viscosity with the properties of RBCs [158]. Still, many considerations need to be taken into account to obtain feasible results.

## 6. Hemorheological Pathologies and Emergent Microfluidics Diagnostics Techniques

As mentioned in previous sections, human blood is an unusual fluid. While blood plasma alone behaves as a Newtonian fluid, the complete set of blood components are non-Newtonian, meaning that, its viscosity varies according to the speed at which it circulates. This characteristic presents two important issues in clinical hemorheology. First, in vitro measurements of the viscosity of plasma alone never reflect the totality of events occurring in vivo in patient circulation. Second, the cellular elements, by acting as particles in suspension, are mainly responsible for the non-Newtonian behavior of blood. This is why, instead of considering only abnormal plasma proteins in diseases, we should also consider the rheological abnormalities of the erythrocytes. The dynamics and the elastic mechanics of RBCs in confined systems are subjects of fundamental interest due to their enormous application potential in biomedical engineering, as they affect hemorheology during blood handling and storage, or manipulate cells in pathology diagnosis. Altered blood due to abnormal RBC concentrations or stiffening of the cells can lead to a reduction in the oxygen delivered or obstruction of the blood vessels. The healthy running of RBC circulation and oxygen transport can be affected by different disorders.

Plasma proteins are responsible for the elevation of blood plasma viscosity in comparison to water; a change in their composition may as well alter the hemorheological properties of blood. Some diseases, such as Wasldenstrom’s macroglobulinaemia, induce an increase in macrogobulins, which increases the viscosity of blood plasma [159]. Furthermore, in this condition, it is more likely that proteins will form rouleaux, which increases blood viscosity at low shear rates. An elevated concentration of fibrinogen in plasma generates an abnormal increase in RBC aggregation, changing the rheological behavior of blood. Aggregation and its effect in blood rheology have been related to several diseases [160,161,162], such as inflammation, diabetes [80,163,164], hypertension [165], obesity [166] and coronary syndromes [167,168,169].

Although plasma affects blood viscosity, RBCs are the most prominent hematological factor influencing hemorheology. Among circulating blood cells, erythrocytes interact most significantly with plasma, mainly as a function of the hematocrit levels. Most typical diseases related to blood viscosity are related to the percentage of RBC concentration (hematocrit), such as anemia (low % hematocrit) or polycythemia (high % hematocrit) [116]. Elevated values of blood viscosity are characteristic of hyperviscosity syndromes. Hyperviscosity may occur due to different properties of blood: an increase in the viscosity of blood plasma, a high production of fibrinogen, an increased numbers of cells (polycythemia or leukemia) or a increased resistance of cells to deformation (sicklemia or spherocytosis) [170,171]. In the case of whole blood, the most influential factor to increase its viscosity is hematocrit. If the hematocrit levels of blood exceed 65% (which is the case of polycythemia), various rheological abnormalities arise, for example, the sedimentation rates decrease significantly as a result of RBC crowding. Additionally, in severe cases, the elasticity and deformation properties of RBCs become crucial to achieving smooth driven flows; otherwise, microcirculation may be severely compromised. On the contrary, anemias present low hematocrit levels, less than 35%. Anemia may have different origins: iron deficiency, hemolysis due to particular diseases or heredity. Hematocrit levels lower than 30% tend to neglect almost every non-Newtonian characteristic and usually displays a Newtonian behavior. However, in some cases of hemolytic anemias, low hematocrit levels lead to high viscosity, due to the alterations of the RBCs’ properties.

Other disorders concern inherited pathologies which affect the RBC membrane, producing abnormalities in RBC shape or deformability, which potentially reduce the healthy functioning of blood circulation. These membrane alterations provide important information about the membrane’s structural balance, and their main causes and consequences are disorders such as sickle cell anemia, hemolytic anemias and thalasemic syndromes, which are directly related to the RBC elasticity, deformation or aggregation properties [16,172]. In addition, some infectious diseases, such as malaria (which does not have a genetic origin), are also known to impair the membrane microstructure, leading to cell stiffening, affecting whole-blood viscosity [173].

Sickle cell anemia (drepanocytosis) is characterized by the formation of sickle cells (ISC) that lose their capability to deform and recover the discocyte shape, altering oxygen delivery. The molecular basis for this is an abnormal phosphorilation of hemogoblin that promotes a massive aggregation of this molecule under low concentrations. The formation of these molecular aggregates affects the concentration of the protein band 3, and the cell membrane is damaged in a process similar to aging, becoming rigid [174]. Patients affected by this pathology present a reduced life expectancy, although modern medical treatments allow a normal life. Due to the presence of ISC, in oxygenated conditions, the ”htc/viscosity” ratio is lower than for normal blood samples. If the natural hematocrit levels of the sample are raised to the typical levels of non-anemic blood, an increased viscosity is observed at all shear rates [82]. The plasma viscosity of subjects with sickle cell anemia is also higher than the viscosity of healthy subjects. In severe cases, ISCs obstruct microvessels, altering the normal circulation of blood. In the last decade, microfluidic devices have played the important role of determining the biophysical characteristics of sickle red cells [175], measuring the mechanical stresses on erythrocytes in sickle cell disease [176], studying vaso-oclusion [104,177,178], identifying biophysical markers [179,180], segregating sickle cells [181], and developing point-of-care diagnostic technologies for low-resource settings [182] and possible treatments [183].

Hemolytic anemias are diseases characterized by the reduction in RBC life expectancy (120 days) and an increased destruction of RBCs (trought hemolysis). Hemolytic disorders originated due to the hereditary defects of three RBC components: the membrane, enzymes and hemoglobin. Hemolysis occurs via two mechanisms, extravascular hemolysis, where RBCs are eliminated prematurely from circulation through the microcirculation fagocitic system (liver and spleen), and intravascular hemolysis, where RBC membranes rupture during blood circulation [184]. In hereditary spherocytosis, patients present a high concentration of spheroidal-shaped RBCs, as a consequence of defects in several proteins of the membrane (mainly from the bilayer–cytoskeleton links), which cause fragility of the membrane. The alteration of membrane properties allows vesiculation and a loss of the membrane surface, triggering cell-shape deformation. Spherocytes rapidly retire from circulation due to the spleenic system, leading to hemolysis. Patients must be treated with blood transfusions for critical levels of anemia [185]. Hereditary elliptocytosis is characterized by abnormalities in the spectrin dimers, causing weakness of the cytoskeleton, which impairs membrane stability. RBCs deform into ellipsoidal (or cigarshaped) cells. The RBC functionality might not be severely affected, as most patients are asymptomatic and only 10% present anemia. Thalassemia syndromes such as αthalassemia and βthalassemia are genetic hematological disorders caused by defects in the synthesis of one or more hemoglobin chains. α-thalassemia, also known as HbH disease, is caused by a reduced or absent synthesis of the α-globin chains, and an excess of β-globin chains in the cytoskeleton. It has been reported [186,187], through measurements of cellular deformability, that α-thalassemic and β-thalassemic erythrocytes exhibit increased surface areas in relation to cell volume, increased membrane rigidity and increased membrane viscosity. Although the stability of α-thalassemic erythrocytes membranes are normal, they are uniformly less dense than healthy erythrocytes. A typical diagnosis for hemolytic anemias is made using a technique known as ektacytometry [188,189]. However, in recent years, microfluidic and lab-on-a-chip devices have presented new alternatives to study RBCs deformation using diverse techniques such as deformability cytometry [103,190], magnetic measurement [191], electrical measurement [192], single-cell chamber arrays [193], combination of microfluidics with machine learning [194] and pressure-driven microrheometry [195].

Malaria is caused by the infection of a parasite of the genus Plasmodium. Infected RBCs develop advanced proteinic machinery, including the formation of organelles similar to the Golgi apparatus, which are used for nutrient transport and storage, and allow enzymatic activity. The parasite is hosted in a vacuole, and during its maturation it reaches the size of a nucleus in a typical eukaryotic cell. Apart from this new internal structure, the parasite produces changes in the membrane proteins that affect the deformability of the cell [196]. RBCs also adopt a more spherical shape, and proteins allocated in the external face of the membrane promote aggregation with other infected cells, avoiding hemolysis in the spleen. All these conformational changes strongly affect the cells’ mechanical properties, modifying the rheological properties of blood [197,198]. As in the case of non-infectious diseases, in recent years, microfluidic devices have been developed to support Malaria diagnosis mostly directed to low-resource locations [199,200,201] or to find possible treatments [202,203,204].

Severe hemorheological disorders are usually related to alterations in the RBCs’ mechanical properties. RBCs have physical properties of their own, and are capable of directly influencing blood flow regardless of hematocrit levels, hence the importance of taking RBCs properties into account when studying its effects on diseases that affects whole blood viscosity. Therefore, understanding these properties from theoretical, numerical, and experimental points of views is the key to improving diagnostics techniques and to developing successful treatments. Microfluidic technologies play a remarkable role in biomedical research, and in combination with biomimetics, lab-on-a-chip and organ-on-a-chip technologies are the cornerstone of future medical diagnoses and treatments.

## 7. Conclusions

In this review, we have described the general features of RBC membranes and their effects on blood flow, from numerical and experimental perspectives, highlighting the achievements of microfluidics technology in performing in vitro studies assessing RBC membranes’ elasticity, hemodynamics and hemorheology.

First, we described the main composition of mammalian cell membranes and refer to the RBC membranes, due to the their special characteristics, specifically their lack of a nucleus and simple structure, which makes them easier to model. We mentioned several methods to model the RBC membranes’ elasticity; however, we discussed the Helfrich free energy model combined with a phase field model to study the cells and vesicles from a single RBC to their collective behavior. In this matter, we found that the dynamics of a single isolated cell immersed in a Poiseuille flow can be modeled using a phase field model coupled with the Navier–Stokes equations. Through this model, we were able to observe the deformation of the RBCs in shear flow.

We later discussed blood and its constitution to relate the elastic properties of the RBCs to the bulk behavior of blood, describing its effect in hemodynamics and hemorheology, again from single-cell to their collective behavior. We presented numerical and experimental points of view considering the latest advances in microfluidics technology to achieve new observations in vitro. From an experimental point of view, we were able to determine the viscosity of blood using microfluidic technology and to determine the effect of RBC aggregation and erythrocyte concentration on whole-blood rheological properties.

Finally, we reviewed and described hematological disorders associated with whole blood and the elastic properties of red blood cells, and how their alterations affect the hemodynamics and rheological properties of blood. We addressed these diseases taking into account the new microfluidic methods that are being developed for diagnostics and future treatment of blood pathologies and their RBC membrane abnormalities. Even though the development of these devices has increased significantly in the past decade, new applications and improvements are being created and discovered every year. Hence, microfluidics applications to diagnostics through the analysis of whole-blood properties or RBCs properties are still and will remain a hot topic in the future.

## Data Availability

All the data is contained within the article.
